# Aging-related changes in reward-based decision-making depend on punishment frequency: An fMRI study

**DOI:** 10.3389/fnagi.2023.1078455

**Published:** 2023-03-06

**Authors:** Ping Ren, Guozhi Luo, Jiayin Huang, Meiling Tan, Donghui Wu, Han Rong

**Affiliations:** ^1^Lab of Brain Health Assessment and Research, Shenzhen Mental Health Center, Shenzhen, Guangdong, China; ^2^Department of Geriatric Psychiatry, Shenzhen Kangning Hospital, Shenzhen, Guangdong, China; ^3^Department of Psychiatry, Shenzhen Kangning Hospital, Shenzhen, Guangdong, China

**Keywords:** decision making, reward, aging, fMRI, frontal subregion

## Abstract

**Introduction:**

Aging is often accompanied by significant cognitive decline and altered decision making. Previous studies have found that older adults have difficulty in processing reward/risk information, leading to suboptimal decision strategy. However, it is still under investigated about the neural substrates of risky decision-making under ambiguity in aging.

**Methods:**

Using the Iowa Gambling Task, the current study investigated inter-individual differences of risk-taking behaviors in healthy older adults with task-related functional magnetic resonance imaging.

**Results:**

It was found that participants were able to improve their decisions in advantageous decks, but failed to avoid disadvantageous decks during task performance. The task-related activations within multiple brain regions were observed significantly different across the four decks, and showed negative correlations with age in disadvantageous decks but not in advantageous decks. Consistently, age-related whole brain analyses confirmed the negative age-effect on brain activations in disadvantageous decks, especially in high punishment frequency. In addition, the relationship between age and task performance in high punishment frequency was mediated by activation in the frontal subregions such as the middle frontal cortex and superior medial frontal cortex.

**Discussion:**

Our findings shed light on the neural substrates of altered risk-taking behaviors in aging, suggesting a greater sensitivity to high punishment frequency in older adults.

## 1. Introduction

Reward-based decision making is critical for survival and development, which is tightly coupled with risk. Individuals need to estimate potential gains and losses to maximize their benefits in response to external stimuli. Aging is often accompanied by declined cognitive function and altered decision making, thereby older adults have greater difficulty in forming optimal decision strategy in uncertain reward and risk conditions (Samanez-Larkin and Knutson, 2015). Although previous studies have reported that older adults exhibit more risk-aversive/conservative behaviors compared with young adults, there are also conflicting results showing no significant difference between the two groups (Mata et al., 2011; Tymula et al., 2013; Rutledge et al., 2016). The inconsistent findings suggest a large inter-individual differences of risk-taking behavior in elderly populations. Therefore, investigating the alterations of reward-based decision making in aging is critical for successful aging and cognitive intervention.

Iowa Gambling Task (IGT) measures risk-taking behaviors in a reward-based reinforcement learning paradigm, which has been extensively used in research and clinical studies (Bechara et al., 1994; Buelow and Suhr, 2009). The classical IGT includes four decks with different frequencies and magnitudes of gain/loss implicitly. During the IGT task, individuals would learn the covert rules and optimize their decision strategies by avoiding disadvantageous decks and choosing advantageous decks. The IGT has been shown to be highly sensitive for testing individual’s decision strategy under ambiguous risk conditions, especially impaired decision making in normal aging and age-related neurodegenerative diseases (Beitz et al., 2014; Sun et al., 2020; Colautti et al., 2021). Compared with young adults, previous studies have reported that older adults stochastically select good and bad decks in the IGT (Fein et al., 2007; Di Rosa et al., 2017). However, other studies have also reported no difference in risk-taking behaviors between the two groups (Wood et al., 2005; Peng et al., 2020). These controversial behavioral findings suggest that more potential factors need to be considered in aging-related alterations of risky decision-making. In most of the IGT studies, task performance usually measured by the difference in long-term selections from advantageous versus disadvantageous decks, and punishment frequency as another important factor has largely been ignored. A series of behavioral studies have reported the “prominent deck B phenomenon” in healthy young adults, showing that participants prefer the disadvantageous deck with low punishment frequency as much as advantageous decks (Dunn et al., 2006; Lin et al., 2007; Arıkan İyilikci and Amado, 2018). Another study has shown that individuals are more sensitive to the effects of long-term reward under the condition of high punishment frequency (Ma et al., 2015). Consistently, Teodorescu et al. recently reported that the combination of high punishment frequency and low fines is more effective in reducing violation behaviors (Teodorescu et al., 2021). Based on these findings, punishment frequency may play a crucial role in understanding the inter-individual differences of risk-taking behaviors in older adults.

So far as we know, only one behavioral study examined the IGT performance corresponding to each deck across the lifespan, showing that deck preferences changed differently as a function of age (Beitz et al., 2014). Using functional magnetic resonance imaging (fMRI), IGT performance have been found associated with multiple brain regions in healthy young adults, including the medial prefrontal cortex (MPFC), inferior parietal lobe (IPL), striatum, and insula (Lin et al., 2008; Li et al., 2010). In healthy older adults, studies have shown a critical role of PFC in processing reward/risk information in different stages of the IGT, suggesting that divergent neurobiological aging trajectories underlie disparate decision-making patterns (Rogalsky et al., 2012; Halfmann et al., 2014). A recent meta-analysis combining IGT and other risky decision-making studies suggested the insula and frontal subregions involved in the task (Tannou et al., 2021). However, there has been less study investigating the neural substrates underlying the influence of punishment frequency on risky decision-making in older adults. In a recent fMRI study, young adults have been found more sensitive to high punishment frequency (deck A), which was closely related to increased neural activity in the anterior cingulate cortex (ACC; Ma et al., 2015). Based on these findings, the neural substrates underlying decision strategies associated with punishment frequency might be changed in aging brain, due to altered loss sensitivity and reduced cognitive efficiency (Samanez-Larkin and Knutson, 2015).

Using the IGT paradigm, the current study investigated the age-related alterations in the neural substrates of risk-taking behaviors corresponding to the four decks in healthy older adults. Our main goals are: (1) to examine the relationship between risk-taking behaviors and punishment frequency in older adults; (2) to identify the brain activations corresponding to different reward/punishment conditions in older adults; and (3) to reveal age-effect on the neural circuits underlying disadvantageous and advantageous options. Taking into account the frequency of punishment, we hypothesized that older adults would still be able to improve their decision strategies during the IGT, and aging might have a greater effect on brain activations associated with disadvantageous decks, especially the high punishment frequency.

## 2. Methods

### 2.1. Participants

Sixty healthy older adults were recruited from multiple communities in Shenzhen. All participants were right-handed determined by the Edinburgh handedness inventory, with adequate visual and auditory acuity for testing by self-report. Exclusion criteria included (1) a history of diagnosed neurological or psychiatric diseases, such as major depression, anxiety, and cerebrovascular disease; (2) MRI contraindications (e.g., metallic implant, claustrophobia, and pacemaker). This study was performed in accordance with the Declaration of Helsinki, and was approved by the Ethics Committee of Shenzhen Kangning Hospital. Each participant was required to sign a written informed consent form after a full written and verbal explanation of the study. Cognitive function was assessed by using the Montreal Cognitive Assessment (MoCA) and Mini Mental State Examination (MMSE), which have been widely used in clinical and research settings to assess multiple cognitive domains (Folstein et al., 1975; Nasreddine et al., 2005; Pinto et al., 2019). Previous studies have shown that older adults with MoCA scores lower than 22 (Chen et al., 2016; Clarnette et al., 2017) or MMSE scores lower than 25 (Feng et al., 2012; An and Liu, 2016) have higher risk for cognitive impairment. However, in order to better examine the relationship between cognitive ability and risk-taking behavior, older individuals with a MoCA score below 22 (ranged from 16 to 29) or a MMSE score below 25 (ranged from 20 to 30) were also included in the current analysis.

### 2.2. Iowa gambling task and behavior analysis

**Figure 1:**
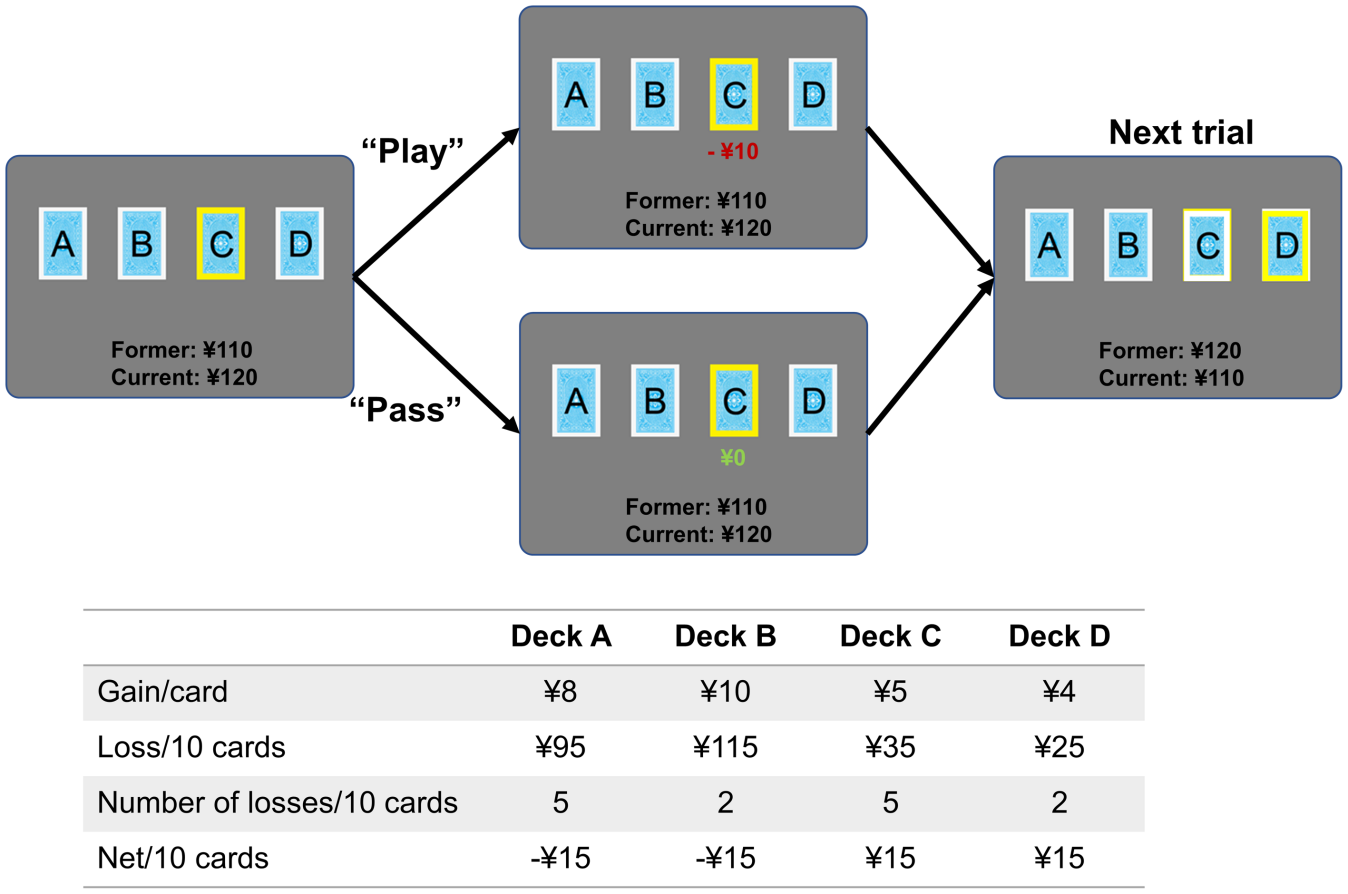
Task paradigm of the Iowa Gambling Task. Each trial began with a yellow frame displayed on one of the four decks randomly. Participants were required to make a response by pressing buttons in the left (“play”) or right (“pass”) hand as soon as possible within 4 s. The jittered inter-trial interval was 2–4 s. The table shows the probability of gain/loss for each deck, and earnings in 10 trials. Cards from decks A and B resulted in higher rewards than cards from decks C and D, but they also resulted in higher losses.

Using an event-related fMRI design, a modified IGT paradigm based on Cauffman and colleague’s version was used during the scanning (Cauffman et al., 2010). At the beginning of each trial, one of the four decks was randomly highlighted with a yellow frame, and participants were required to make a decision whether to play or pass the card as soon as possible. Participants were informed that with each selected card (“play”) they would win or lose money (a net gain/loss) based on different probability, and with each unselected card they would not win or lose any money. Different from the standard IGT, our customized IGT paradigm was modified in punishment frequency and net gain to slightly increase task difficulty (Figure 1). Decks A and B were defined as bad decks with high net gains (¥8 and ¥10) but long-term loss (¥15 loss per 10 cards), while decks C and D were defined as good decks with low net gain (¥5 and ¥4) but long-term reward (¥15 gain per 10 cards). Decks A and C resulted in losses on 50% of selections, whereas Decks B and D resulted in losses on 20% of selections. A participant might select a card that contains only a win, or select a card that contains a win plus a loss. The payoff ranges were from −¥12 to ¥8 for deck A, from −¥50 to ¥10 for deck B, from −¥3 to ¥5 for deck C, and from −¥9 to ¥4 for deck d. Therefore, choosing from the low-risk decks (deck C and D) was advantageous choice, while choosing from the high-risk decks (deck A and B) was disadvantageous choice. With a bank of ¥100 at the beginning of the IGT, participants were required to earn monetary reward as much as possible. For each trial, participants need to make a response within 4 s, then a feedback was displayed showing the corresponding gain or loss. Trials were separated by an inter-trial interval jittered between 2 and 4 s. The total number of trials in the entire task was between 80 and 85, with at least 20 trials for each deck. After the entire experiment, participant would be paid actual money (¥50–¥200) based on their performance.

For each deck, deck choice (proportion of selections) and total income (final earnings) were computed to measure individual’s risk preference. To examine the task performance over time, the entire task was divided into four blocks with ~20 trials per block (number of trials was dependent on participants’ performances). To measure the change of decision strategy during the IGT, change of deck selection (CDS) was defined by calculating the difference of deck choice between the last and first blocks (CDS = Deck_choice*_block4_* – Deck_choice*_block1_*). Therefore, a positive CDS indicated increased number of selection, and a negative CDS indicated decreased number of selection.

### 2.3. Imaging data acquisition

Neuroimaging data were acquired on a 3.0 T MRI system (Discovery MR750 System, GE Healthcare) with an eight-channel phased-array head coil. The T1-weighted images were acquired by using a three-dimensional brain volume imaging sequence that covered the whole brain (repetition time (TR) = 6.7 ms, echo time (TE) = 2.9 ms, flip angle = 7 degrees, matrix = 256 × 256, slice thickness = 1 mm, 196 slices). In the task-related fMRI, a gradient-echo echo-planar imaging sequence was used with the following parameters: TR = 2,000 ms, TE = 25 ms, flip angle = 90 degrees, matrix = 64 × 64, and voxel size = 3.4 × 3.4 × 3.2 mm^3^, and 48 axial slices. During the task session, participants made responses by pressing left-handed button for play and right-handed button for pass. The entire task lasted 10 min with 300 volumes.

### 2.4. Functional imaging data preprocessing

The functional images were preprocessed in the DPABI_v4.3^1^ (Yan et al., 2016) based on the SPM8.^2^ The first 5 volumes were excluded to obtain steady-state tissue magnetization. The remaining volumes were corrected for slice timing and head motion, co-registered to structural images, and normalized to the Montreal Neurological Institute (MNI) standard space. Then the functional images were resampled to 3 × 3 × 3 mm, and smoothed with a Gaussian kernel (FWHM = 6 mm). After preprocessing, six older adults were removed from the formal analysis due to head motion >2.5 mm or 2.5 degrees. Compared with young adults, older adults often have larger head motion, which has been known to have significant confounding effects on resting-state functional connectivity and task-related brain activity (Van Dijk et al., 2012; Siegel et al., 2014). Therefore, the average displacement of head motion was calculated (displacement = square root (*x*^2^ + *y*^2^ + *z*^2^); *x*, left/right; *y*, anterior/posterior; *z*, superior/inferior), and further controlled as a covariate in the following analyses (McDermott et al., 2019; Ren et al., 2020).

### 2.5. Voxel-based morphometry

The T1-weighted images were preprocessed to generate a whole-brain gray matter (GM) map using Voxel-based morphometry (VBM). Each individual’s T1-weighted image was segmented into gray matter, white matter, and cerebrospinal fluid. Then, a gray matter template was generated through an iterative nonlinear registration using DARTEL, a toolbox with a fast diffeomorphic registration algorithm (Ashburner, 2007). For each participant, an averaged gray matter of the entire brain was computed to control age-related GM atrophy in the following analyses.

### 2.6. Task-related brain activation analysis

A general linear model was applied to examine brain activations linked to four event-related regressors, including the deck A, B, C, D. The event onset is the time point when a yellow frame is on. Since the current study focused on the neural substrates of decision process for each deck in aging, both play and pass conditions represent individual’s decision strategy and learning process. Therefore, these two conditions were not added as regressors following the same way that previous IGT studies did (Lawrence et al., 2009; Ma et al., 2015). Specifically, the regressors were generated by convolving the onset times of each type of events with a canonical hemodynamic response function (HRF). The model also included non-interested regressors as covariates in the design matrix, including six head motion parameters, CSF and white matter signals. The regressors were convolved with a canonical hemodynamic response function. The trials with no response were excluded, and a high-pass filter with a cut-off period of 128 s was used to remove slow drift.

For each participant, contrast images were generated to examine the brain activations corresponding to decks A, B, C, and D, respectively. A one-way repeated measures ANOVA was applied to examine differences of brain activations across the four decks. To further examine age-effect on brain activation, a partial correlation was performed to generate correlation map for each deck, controlled for MoCA, head motion, and GM. The resulting statistical maps were corrected for multiple comparison using FDR corrected *p* < 0.05 and cluster >50 voxels. Notably, using the combination of a value of *p* and a cluster size is a general way to avoid Type I error. Previous studies often used a threshold of FDR corrected *p* < 0.05 with arbitrary cluster criterion, such as 5 voxels, 10 voxels, and 50 voxels (Keulers et al., 2012; Ortiz-Rios et al., 2017; Bhandari et al., 2020). Here we mainly focused on the core regions associated with task conditions, therefore, a more conservative cluster criterion (>50 voxels) was used to reduce false positives and exclude less important brain regions. Regions of interest (ROIs) were defined as 6 mm radius spheres centered in the peaks, and averaged brain activations were extracted to do the following correlation and mediation analyses. Based on the ROIs defined in the ANOVA, a coefficient matrix was generated to compare the age-effects across the four decks by calculating partial correction between age and brain activation. Subsequently, a voxel-wise partial correlation was performed between age and brain activation in each deck to examine the age-effects across the entire brain (FDR corrected *p* < 0.05, cluster >50 voxels). Brain activations were extracted as mentioned above, then a partial correlation was used to examine the relationship between IGT performance and brain activation, controlled for MoCA, GM, and head motion. Here the partial correlations between brain activation and age/behavior have been controlled for MoCA, GM, and head motion.

### 2.7. Other statistical analysis

Other statistical analyses were conducted in Matlab 2014b and SPSS V22. A mediation analysis was used to examine whether age influence task performance through brain region in older adults (number of bootstrap samples = 5,000) using the PROCESS macro in the SPSS (Hayes, 2017). In the mediation model, path a represents the relationship between independent variable X (age) and mediator (brain activity), and path b represents the relationship between mediator (brain activity) and dependent variable Y (CDS of deck A). The chief estimated path is the total effect of X on Y (i.e., path c). This path comprises the direct effect of X on Y after controlling for the (i.e., path c’) and the indirect effect of X on Y through the mediator (i.e., path a × b). After accounting for the mediator, the mediation effect was determined by assessing whether there was a significant difference between the total effect (i.e., path c) and direct effect (i.e., path c’). In the mediation models, non-interested covariates were controlled as confounding factors, including MoCA, GM, and head motion.

## 3. Results

### 3.1. Behavioral performance in the IGT

**Figure 2:**
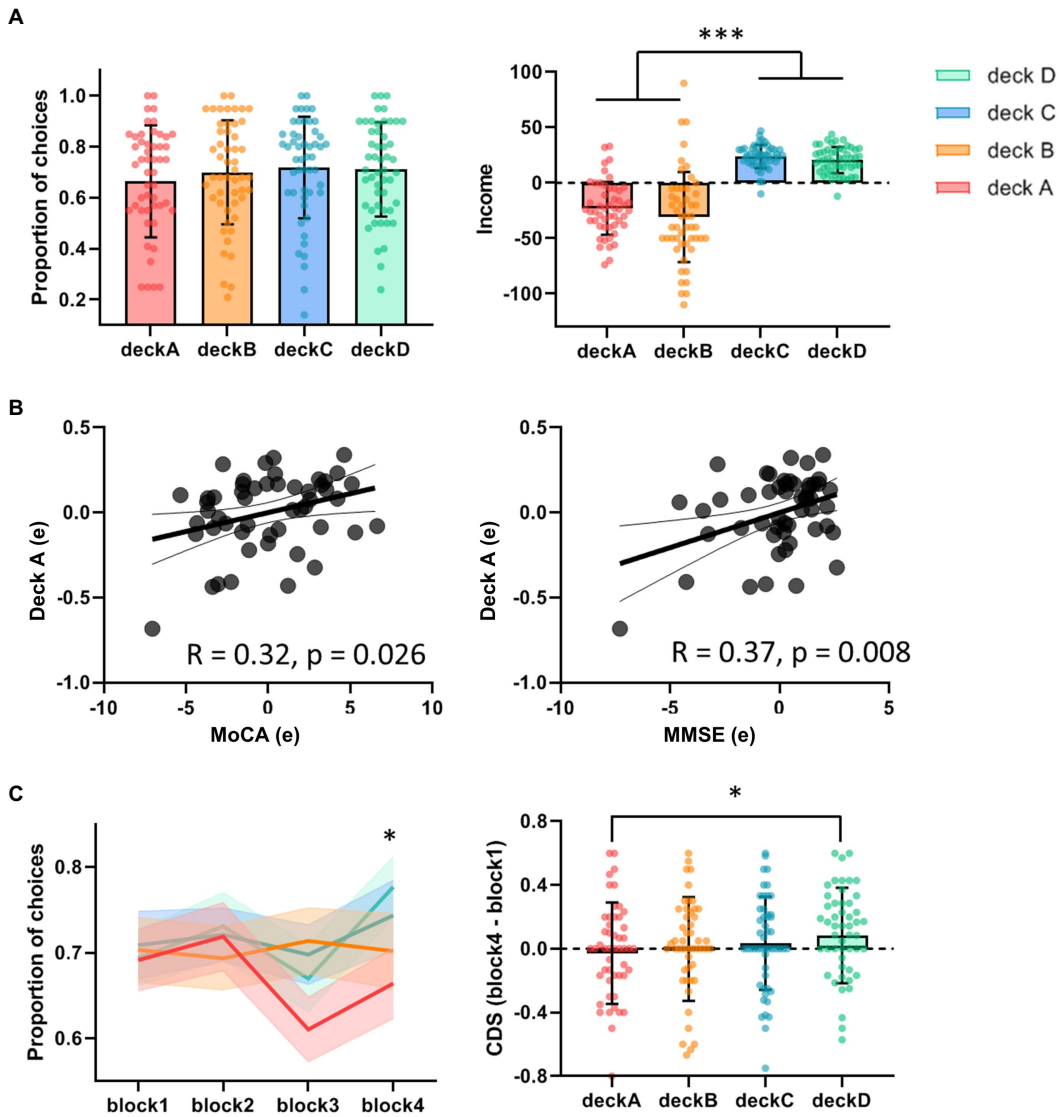
Task performance in the IGT. **(A)** The proportions of deck selection showed no significant difference across the four decks. The total incomes were significantly higher in advantageous decks versus disadvantageous decks. **(B)** Partial correlations showed positive correlations between deck A choice and MoCA/MMSE, controlled for age and education. **(C)** In the block analyses, older adults tended to avoid choosing deck A, showing significant difference of CDS between deck A and deck D. *p < 0.05; ***p < 0.005.

Before behavior data analyses, three participants were removed from the analysis due to inappropriate task performance (i.e., they played or passed all cards on the four decks). The overall task performances (i.e., advantageous choice, disadvantageous choice, and advantageous versus disadvantageous choice) were not significantly correlated with age, education, MMSE, MoCA, GM, or head motion. Total income in the IGT was negatively correlated with disadvantageous choice (bad decks) and positively correlated with advantageous vs. disadvantageous choices (good versus bad decks; Table 1). Older adults showed slightly higher proportion of choices in good decks (0.72 ± 0.17) relative to bad decks (0.68 ± 0.19), however, the difference between good and bad decks were not significant (*t* = 1.32, *p* = 0.19; Table 1; Figure 2A). Participants showed significantly higher task income in decks C and D compared with decks A and B (*t* = 12.80, *p* < 0.001). Partial correlation analyses showed that deck A choice was positively correlated with MoCA and MMSE, controlled for age and education (Figure 2B). If correcting multiple comparison by FDR *p* < 0.05, deck A choice was still positively correlated with MMSE (*p* = 0.032), but not with MoCA (*p* = 0.10). In the block analyses, a paired *t*-test was conducted to examine the selection differences between disadvantageous and advantageous choices in four pairs of conditions: deck A versus deck C, deck A versus deck D, deck B versus deck C, and deck C versus deck D. The results showed that participants selected less deck A than deck D (*t* = −3.07, FDR corrected *p* = 0.012) in the last block, and no other difference was observed (Supplementary Table 1). Likewise, CDS was significantly different in the condition of deck A versus deck D (*t* = −2.58, *p* = 0.040; Figure 2C), but not in the conditions of deck A versus deck C (*t* = −1.15, *p* = 0.33), deck B versus deck C (*t* = −0.66, *p* = 0.51), or deck B versus deck D (*t* = −1.51, *p* = 0.28), with FDR correction. In addition, a one sample *t*-test was used to determine whether the CDS values were different from zero in decks A and D. The results showed marginally significant difference for deck D (*t* = 1.98, *p* = 0.053), and no significance for deck A (*t* = −0.61, *p* = 0.55). The behavioral analyses indicated that older adults might be still able to learn better decision strategy in the IGT.

**Table 1:** Demographic, neuropsychological assessments and IGT performance.

	*N* = 51	Good	Bad	Good vs. Bad
Age	64.29 ± 5.44	0.008	−0.10	0.12
Male/Female	26/25	–	–	–
Education (years)	11.91 ± 2.37	−0.31	−0.070	−0.22
MOCA	22.51 ± 3.38	−0.016	0.12	−0.16
MMSE	27.78 ± 2.03	−0.051	0.21	−0.28
Global GM	0.31 ± 0.02	−0.03	−0.20	−0.24
Head motion	1.11 ± 0.81	−0.12	−0.10	0.00
Good	0.71 ± 0.17	–	–	–
Bad	0.68 ± 0.19	0.55***	–	–
Good vs. Bad	0.032 ± 0.17	0.39**	−0.56***	–
Income	89.75 ± 46.88	−0.033	−0.38**	0.38**

Data are presented as means ± standard deviations for the entire sample. Pearson correlation was applied to examine the relationships between IGT performance and other measurements. **p < 0.01; ***p < 0.005. GM, gray matter; IGT, Iowa gambling task; MOCA, Montreal Cognitive Assessment; MMSE, Mini Mental State Examination.

### 3.2. Task-related differences in brain activations corresponding to the four decks

**Figure 3:**
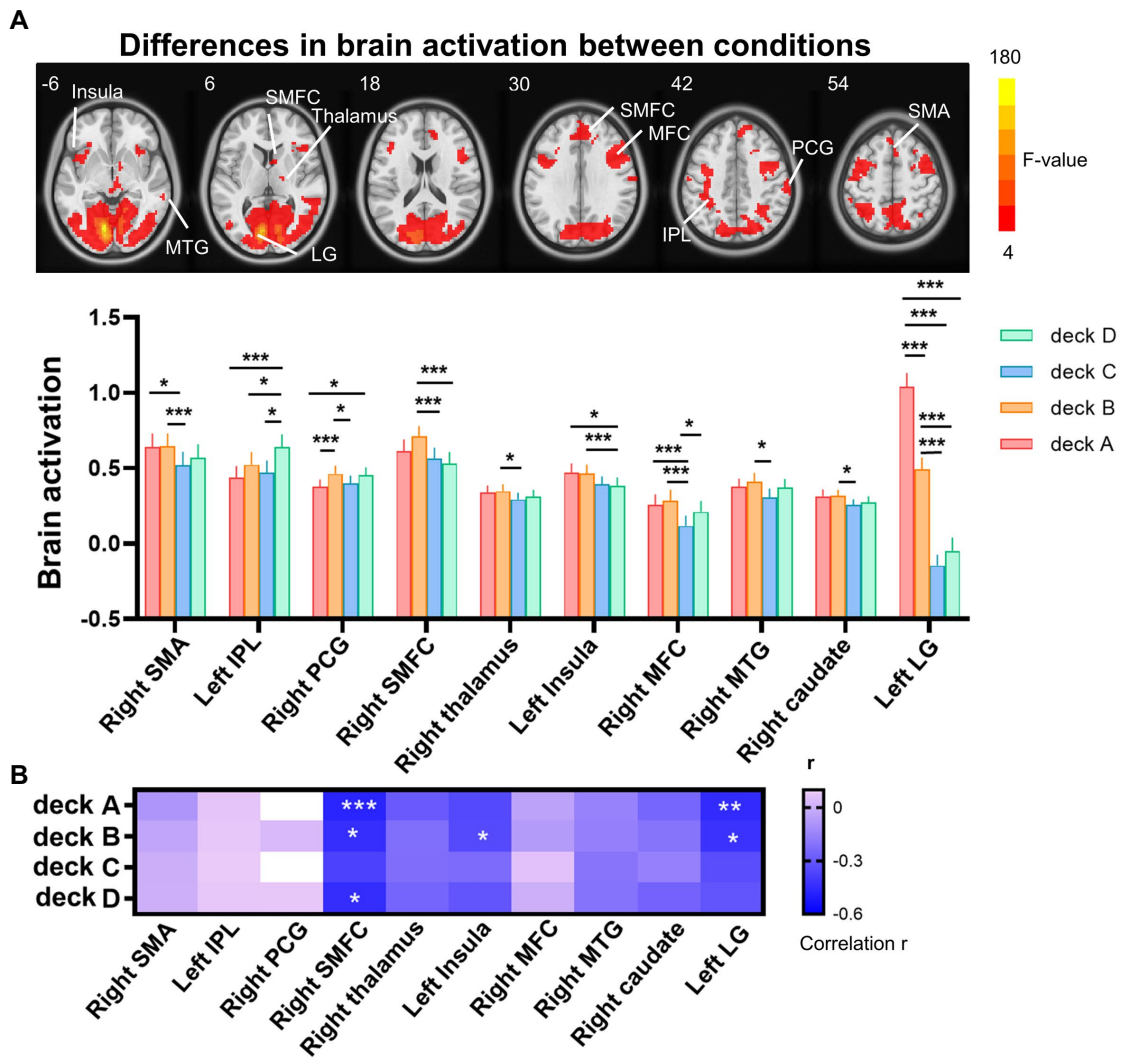
Differences of brain activation across the four decks during the IGT. **(A)** The one-way repeated measures ANOVA showed significant differences of brain activation corresponding to the four decks in the right SMA, left IPL, right PCG, right SMFC, right thalamus, left insula, right MFC, right MTG, right caudate, and left LG. **(B)** For each deck, significant correlations between brain activation and age were labeled with asterisks, controlled for MoCA, GM, and head motion (FDR corrected *p* < 0.05). The heat map of partial correlation coefficients exhibits that age-effect was mainly in disadvantageous decks relative to advantageous decks. IPL, inferior parietal lobe; LG, lingual gyrus; MFC, middle frontal cortex; MTG, middle temporal gyrus; PCG, postcentral gyrus; SMA, supplementary motor area; SMFC, superior medial frontal cortex. *p < 0.05; **p < 0.01; ***p < 0.005.

The one-way repeated measures ANOVA was performed to examine differences of brain activations corresponding to the four decks. The results showed significantly differences in the right supplementary motor area (SMA), left inferior parietal lobe (IPL), right postcentral gyrus (PCG), right thalamus, left insula, right middle frontal cortex (MFC), right middle temporal gyrus (MTG), right caudate, and left lingual gyrus (LG; Table 2; Figure 3A). For visualization purpose, the averaged brain activations within each ROI were extracted to show the differences across the four decks as well. The coefficient matrix showed partial correlations between age and brain activations for each deck, showing greater correlations in disadvantageous decks, especially in deck A with high punishment frequency (Figure 3B; Supplementary Figures 1, 2). Additional analyses were performed to examine the differences in task-related brain activation between each pair of decks in Table 3 and Supplementary Figure 3. The results showed significant brain regions consistent with the findings in the ANOVA.

**Table 2:** Differences of task-related brain activation across the four decks.

Region	*F* value	Cluster (voxels)	MNI coordinates	BA
Right SMA	9.44	124	3, 9, 63	6/8
Left IPL	15.41	850	−33, −51, 51	6/40/7
Right PCG	8.50	78	63, −18, 45	4
Right SMFC	13.64	278	6, 42, 30	9/32
Right thalamus	6.80	53	18, −12, 3	N/A
Left insula	8.86	60	−30, 21, −9	47
Right MFC	16.66	947	45, −3, 51	6/9/47
Right MTG	7.51	61	48, −15, −15	N/A
Right caudate	7.20	109	9, 3, −6	N/A
Left LG	187.47	7,688	−9, −75, −6	18/7/19

A one-way Repeated measures ANOVA was performed to examine the differences of brain activation across the four decks during the IGT (FDR corrected *p* < 0.05, cluster > 50 voxels). BA, Brodmann area; IPL, inferior parietal lobe; LG, lingual gyrus; MFC, middle frontal cortex; MTG, middle temporal gyrus; PCG, postcentral gyrus; SMA, supplementary motor area; SMFC, superior medial frontal cortex.

**Table 3:** The differences of brain activity between each pair of decks.

Region	*t* value	Cluster (voxels)	MNI coordinates	BA
Deck A vs. Deck B				
Left LG	14.13	1943	−6, −78, 3	18/19/6
Right LG	−7.95	60	9, −78, −12	18
Right Mid_OC	−7.96	181	27, −90, 3	18
Deck A vs. Deck C				
Left precuneus	6.31	128	0, −60, 60	7
Right MFC	5.67	69	42, −3, 51	6
Right LG	−8.41	181	15, −96, 3	18/17
Left LG	18.43	3,063	−9, −72, −9	18/6/19
Deck A vs. Deck D				
Left IPL	−5.75	108	−33, −51, 51	40/7
Right LG	5.96	52	21, −54, 0	19/30
Left OC	−5.68	74	−45, −66, −3	37
Right calcarine	−11.56	494	12, −81, 3	18/6/17
Left LG	15.82	861	−9, −84, 0	18/6/17
Deck B vs. Deck C				
Left MFC	5.67	229	−42, 3, 57	6/9
Left ACC	4.95	95	0, 39, 24	9/32
Right cuneus	−6.98	95	15, −96, 9	18/17
Right LG	5.60	58	18, −54, −3	N/A
Right MFC	7.37	652	45, −3, 51	6/9/47
Right OC	6.77	1,106	45, −66, −15	19/7/37
Left LG	14.88	2,267	−9, −75, −6	6/18/19
Deck B vs. Deck D				
Right SMFC	6.49	63	6, 45, 30	9
Right calcarine	−12.82	688	12, −81, 3	18/19/17
Right Mid_OC	7.57	297	30, −90, 0	18/19/37
Left Mid_OC	11.29	676	−18, −99, 6	18/6/17
Deck C vs. Deck D				
Left IPL	−5.46	81	−33, −48, 51	7
Left Mid_OC	7.54	57	−15, −102, 0	N/A
Right LG	−13.93	2,639	12, −66, −6	18/7/19

A paired *t*-test examined the activity differences between each pair of decks during the IGT (FDR corrected *p* < 0.008, cluster > 50 voxels). BA, Brodmann area; ACC, anterior cingulate cortex; IPL, inferior parietal lobe; LG, lingual gyrus; MFC, middle frontal cortex; MTG, middle temporal gyrus; OC, occipital cortex; PCG, postcentral gyrus; SMA, supplementary motor area; SMFC, superior medial frontal cortex.

### 3.3. Age-effect on brain activations corresponding to the four decks

**Figure 4:**
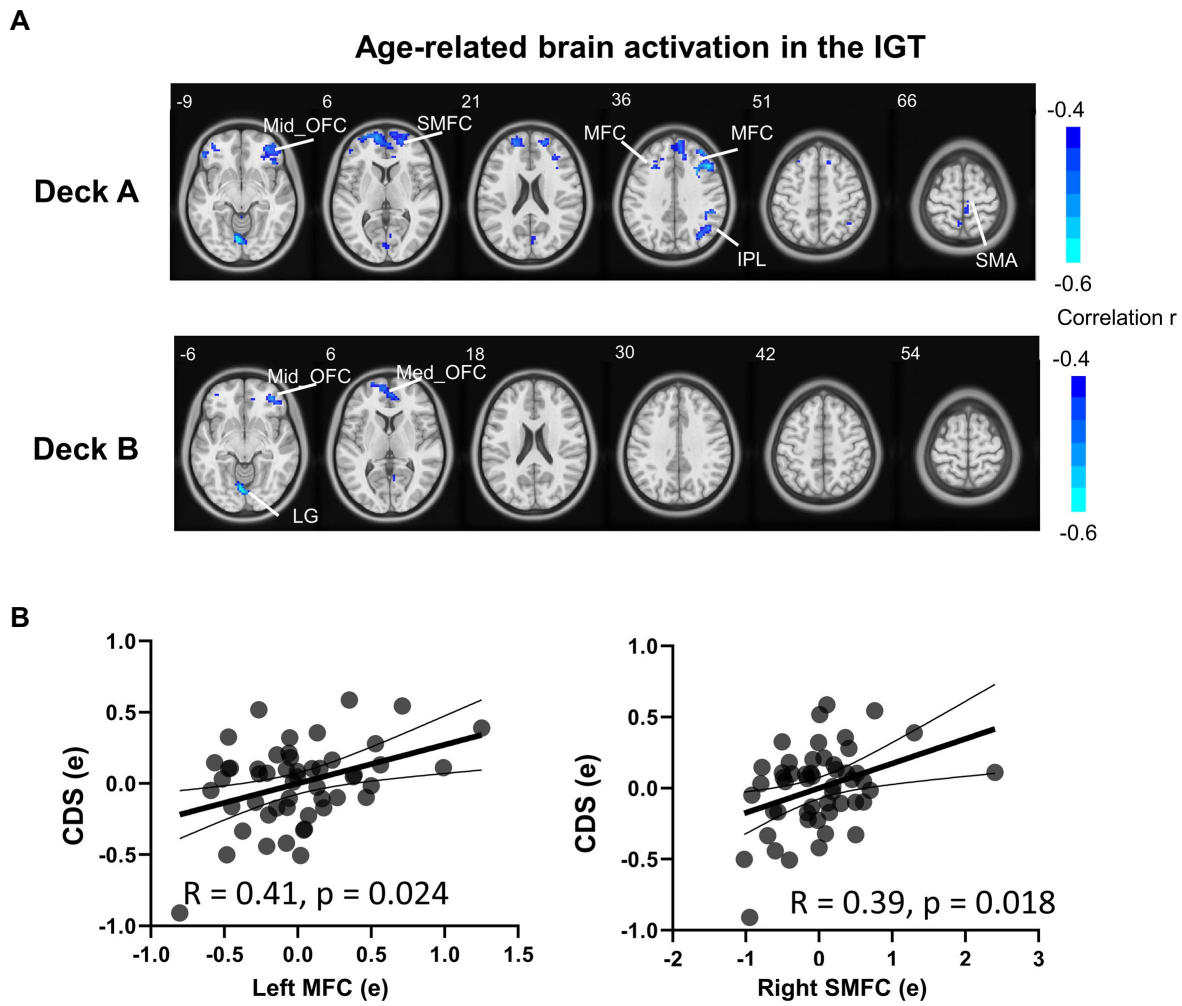
Brain activation associated with age during the IGT. **(A)** The results showed significantly negative correlations between age and brain activation in multiple brain regions in disadvantageous decks, but not in advantageous decks. **(B)** In the condition of deck A, the brain activations were significantly correlated with CDS in the left MFC and right SMFC (FDR corrected *p* < 0.05). The correlation analyses were controlled for MoCA, head motion, and GM. MFC, middle frontal cortex; SMFC, superior medial frontal cortex.

To examine age-related brain activation maps during the IGT, age was correlated to brain activations corresponding to the four decks, respectively (Table 4; Figure 4A). In deck A, age was negatively associated with brain activations in the right SMA, right IPL, right superior medial frontal cortex (SMFC), right middle orbitofrontal cortex (OFC), and bilateral MFC. In deck B, age was negatively associated with brain activations in the medial OFC, right middle OFC, and lingual gyrus (LG; Table 4; Figure 4A). There was no significant correlation in the conditions of decks C and D. For each significant brain region, brain activation was extracted and associated with task performance, showing that CDS of deck A was positively correlated with brain activations in the left MFC (r = 0.41, *p* = 0.024) and right SMFC (r = 0.39, *p* = 0.018) with FDR correction (Figure 4B). There was no significant correlation between CDS and brain activation in other regions (Supplementary Table 3).

**Table 4:** Age-effect on brain activations during the IGT.

Region	*t* value	Correlation coefficient	Cluster (voxels)	MNI coordinates	BA
Deck A					
Right SMA	−4.63	−0.56	112	6, −36, 69	6/4
Right IPL	−5.01	−0.59	217	33, −72, 42	40
Left MFC	−5.42	−0.62	134	−27, 30, 45	8
Right MFC	−5.71	−0.64	291	42, 24, 39	9
Right SMFC	−4.51	−0.55	720	6, 45, 30	10/9
Right Mid_OFC	−4.17	−0.52	134	33, 45, −15	11
Deck B					
Med_OFC	−4.63	−0.56	144	0, 54, −3	10
Right Mid_OFC	−4.63	−0.56	54	36, 45, −9	N/A
LG	−5.71	−0.64	117	0, −78, −6	N/A

A voxel-wise partial correlation analysis was performed between age and brain activation in each deck, controlled for MoCA, GM, and head motion (FDR corrected *p* < 0.05, cluster > 50 voxels). BA, Brodmann area; IPL, inferior parietal lobe; SMFC, superior medial frontal cortex; LG, lingual gyrus; MFC, middle frontal cortex; Mid_OFC, middle orbitofrontal cortex; Med_OFC, medial orbitofrontal cortex; SMA, supplementary motor area.

### 3.4. Mediation analysis

**Figure 5:**
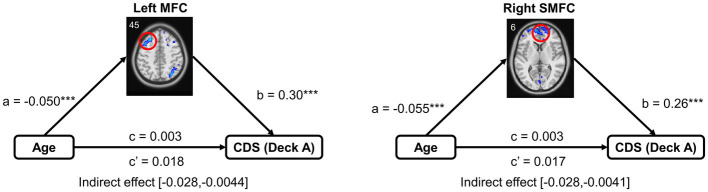
Mediation analysis in the relationship between age and task performance. Mediation analysis showed that brain activation of the frontal subregions (the left MFC and right SMFC) mediated the relationship between age and CDS in high punishment frequency, controlled for MoCA, GM, and head motion. MFC, middle frontal cortex; SMFC, superior medial frontal cortex. ***p < 0.005.

A Mediation model was performed to examine brain activation in mediating the relationship between age and IGT performance, controlled for MoCA, GM, and head motion. Only the left MFC and right SMFC in deck A were selected to do the mediation analysis because they were significantly correlated to both age and CDS. In older adults, left MFC activity significantly mediated the relationship between age and CDS of deck A (indirect effect = −0.015, SE = 0.006, 95% CI [−0.028, −0.0044]). Right SMFC activity significantly mediated the relationship between age and CDS of deck A (indirect effect = −0.014, SE = 0.006, 95% CI [−0.028, −0.0041]). The details of the mediation analyses were displayed in Figure 5.

## 4. Discussion

In the current study, we investigated the neural correlates of individual differences in risk-taking behavior in older adults combining IGT performance and task-related fMRI data. To the best of our knowledge, this is the first study examining age-effect on brain activations in response to different punishment frequencies in older adults. The brain activations corresponding to the four decks were significantly different in widespread brain regions. However, the correlations between age and brain activity were observed mainly in the condition of disadvantageous decks, showing that age-related changes in brain activity were more widespread in high punishment frequency. Specifically, increased age was associated with reduced brain activations in the frontal subregions, parietal lobe and lingual gyrus. Additionally, the relationship between age and task performance in high punishment frequency was mediated by activations in the MFC and SMFC. These findings indicated that neural circuits underlying disadvantageous options were remarkably changed in the aging brain, suggesting a critical role of punishment frequency in altered decision making in older adults.

Different from those studies comparing older adults to young adults, here we focused on age-effect on the brain circuits underlying altered risk-taking behaviors in older adults. The current analyses preclude the confounding factors of cohort effects and age-related brain anatomical/functional disparities in fMRI studies on aging (Samanez-Larkin and D’Esposito, 2008; Halfmann et al., 2014, 2016). Our results showed no significant difference of overall selections across the four decks, supporting previous findings that older adults have difficulty in forming optimal decision strategy in the IGT (Fein et al., 2007; Di Rosa et al., 2017). However, older adults learned to select more good decks over time, and exhibited significant difference between deck A and deck D in the last block. The results showed that selections from deck A was positively associated with cognitive scores, in line with previous studies showing individuals with higher cognitive ability are more patient and willing to take more risks (Dohmen et al., 2010; Deck and Jahedi, 2015). These findings suggest that older adults are still capable of distinguishing advantageous and disadvantageous options at some level, however, age-related cognitive decline might influence risk-taking behavior in the condition of high frequency punishment.

In the task-related fMRI data analyses, brain activations corresponding to the four decks were observed significantly different in widespread brain regions. Compared with advantageous decks, brain activations correlated to disadvantageous decks were higher in the MFC, SMFC, temporal and subcortical regions, and lower in the parietal lobe. Consistently, previous studies have reported brain activation during choices from disadvantageous versus advantageous decks mainly in the ACC/SMFC, MTG, and thalamus (Ma et al., 2015). It has long been known that the MTG is the hub of the default mode network (DMN; Smallwood et al., 2021), and the SMFC, MFC and IPL are the hubs of the frontoparietal network (FPN; Harding et al., 2015; Scolari et al., 2015). Bolt et al. have reported that global and regional network organization is significantly modulated across states during the IGT, emphasizing the critical roles of the DMN and FPN across task states (Bolt et al., 2016). Likewise, our findings suggest that the two functional networks are of great importance for successful IGT performance. Interestingly, a significantly higher activity was observed in the lingual gyrus/occipital cortex in the conditions of disadvantageous decks, especially deck A with high punishment frequency. Here increased activation in the primary sensory cortex might be representative of an enhanced level of attention or arousal, in line with the adolescent study showing greater occipital activation in high versus moderate levels of risk/reward (Morales et al., 2018). More importantly, individuals exhibited significantly negative correlations between age and brain activations in the SMFC, insula and lingual gyrus in disadvantageous decks, especially in high punishment frequency (deck A). These findings suggest that the distinct brain activations in different reward/punishment conditions might underlie the individual differences of task performances in older adults, showing more sensitive to disadvantageous options with high punishment frequency in aging brain.

In the further analyses, voxel-based whole brain analysis showed significantly negative age-effect on brain activations in disadvantageous decks but not in advantageous decks. Specifically, increased age was correlated with reduced activity in the SMA, MFC, SMFC, OFC, and LG, which confirmed our findings in the ANOVA. Consistently, recent studies reported a critical role of the OFC/vmPFC and dorsolateral PFC in modulating young and older adults’ IGT performance (Rogalsky et al., 2012; Tannou et al., 2021; Zha et al., 2022a). Furthermore, brain activations in the MFC and SMFC were positively correlated with CDS, and mediated the relationships between age and task performance in high punishment frequency. The mediation model showed that activations in the frontal subregions supports indirect relationships between age and risk-taking behaviors in older adults. Our results suggest that the MFC and SMFC might be crucial for understanding the individual differences of risk-taking behaviors in aging. In line with our findings, previous findings have shown that MFC played an critical role in a reinforcement learning model in the IGT (d'Acremont et al., 2009). Other studies have shown that the SMFC is significantly activated during disadvantageous versus advantageous choices in the IGT (Lawrence et al., 2009; Ding et al., 2017), and during difficult versus easy decisions in the delay discounting task (Zha et al., 2022b). Therefore, the negative correlations between age and the frontal activations in disadvantageous decks might lead to cognitive inefficiency and emotion dysregulation in aging brain, which leads to sub-optimal decision strategy in older adults.

Several limitations of our study must be acknowledged. First, the current study did not include young counterparts, thus we compared our behavioral data with previous studies with young adults. Future research needs to validate the similarity and difference in brain activation patterns between older and young groups. Second, the current analyses included older adults with a large range of MoCA/MMSE scores, in order to better examine the relationship between cognitive decline and risk-taking behaviors. Those participants with low cognitive scores might have potential cognitive deficits or high risk of mild cognitive impairment (MCI). However, it is still debatable whether normal aging and MCI are significantly different in reward-based decision making (Pertl et al., 2015; Coelho et al., 2017; Sun et al., 2020). These controversial findings suggest that it is important to further examine the relationship between cognitive ability and risky decision-making in future studies. In addition, previous studies have reported significant differences between males and females in the IGT performance (van den Bos et al., 2013). However, our additional analysis showed no gender differences in the IGT performance (Supplementary Table 2), which might be due to large individual differences in older adults and/or small sample size in the current study. Future research should involve a large cohort to investigate gender effect in aging.

In summary, the current study found a considerable age-effect on neural correlates of reward-based decision making in older adults. Compared with advantageous options, aging induced widespread changes of brain activations corresponding to disadvantageous options, especially the high punishment frequency. Our findings would allow us to better disentangle the individual differences of reward-based decision making in aging, suggesting a greater sensitivity to high punishment frequency in older adults.

## Data availability statement

The raw data supporting the conclusions of this article will be made available by the authors, without undue reservation.

## Ethics statement

The studies involving human participants were reviewed and approved by the Ethics Committee of Shenzhen Kangning Hospital. The patients/participants provided their written informed consent to participate in this study.

## Author contributions

PR and GL designed the experiment and wrote the manuscript. PR analyzed the data. JH and MT collected data. DW and HR helped improve the manuscript. All authors contributed to the article and approved the submitted version.

## Funding

This work was supported by Natural Science Foundation of Guangdong Province 2020A1515011526 to PR, Shenzhen Science and Technology Innovation Commission JCYJ20210324133208023 to PR, Sanming Project of Medicine in Shenzhen SZSM201812052, Guangdong Natural Science Foundation for Major Cultivation Project 2018B030336001, Natural Science Foundation of Guangdong Province 2021A1515011152, and Shenzhen Fund for Guangdong Provincial High-level Clinical Key Specialties SZGSP013.

## Conflict of interest

The authors declare that the research was conducted in the absence of any commercial or financial relationships that could be construed as a potential conflict of interest.

## Publisher’s note

All claims expressed in this article are solely those of the authors and do not necessarily represent those of their affiliated organizations, or those of the publisher, the editors and the reviewers. Any product that may be evaluated in this article, or claim that may be made by its manufacturer, is not guaranteed or endorsed by the publisher.

## Footnotes

^1^ https://rfmri.org/dpabi

^2^ http://www.fil.ion.ucl.ac.uk/spm/
