# Supplementary material for: Aging-related changes in reward-based decision-making depend on punishment frequency: An fMRI study
Source: Front Aging Neurosci. 2023 Mar 6;15:1078455. doi: 10.3389/fnagi.2023.1078455 (PMC10025509; doi:10.3389/fnagi.2023.1078455)

| Supplementary Table 1. Selection differences between disadvantageous and advantageous decks in each block. | | | | |
| --- | --- | --- | --- | --- |
|  | Block 1 | Block 2 | Block3 | Block4 |
| Deck A vs. Deck C | -0.41 | -0.05 | -1.99 | -1.75 |
| Deck A vs. Deck D | -0.06 | -0.27 | -1.26 | **-3.07*** |
| Deck B vs. Deck C | -0.13 | -0.69 | 0.42 | -0.22 |
| Deck B vs. Deck D | 0.20 | -0.79 | 0.91 | -0.16 |

A paired t-test was conducted to examine the selection differences between disadvantageous and advantageous decks in each block with FDR correction. Statistical t value was displayed for each comparison. *, FDR corrected p<0.05.

**Supplementary Table 2. Gender differences in the IGT performance.**

|  | Male (N = 26) | Female (N = 25) | p value |
| --- | --- | --- | --- |
| Good | 0.75 ± 0.14 | 0.68 ± 0.20 | 0.19 |
| Bad | 0.72 ± 0.18 | 0.64 ± 0.20 | 0.15 |
| Good vs. Bad | 0.025 ± 0.15 | 0.039 ± 0.19 | 0.78 |
| Income | 88.75 ± 43.49 | 90.78 ± 51.04 | 0.88 |
| *Proportion of choices* |  |  |  |
| Deck A | 0.73 ± 0.17 | 0.60 ± 0.25 | 0.15 |
| Deck B | 0.71 ± 0.21 | 0.69 ± 0.20 | 0.64 |
| Deck C | 0.75 ± 0.16 | 0.69 ± 0.23 | 0.33 |
| Deck D | 0.74 ± 0.16 | 0.68 ± 0.21 | 0.33 |
| *CDS* |  |  |  |
| Deck A | 0.034 ± 0.0.37 | -0.091 ± 0.25 | 0.66 |
| Deck B | -0.037 ± 0.32 | 0.036 ± 0.34 | 0.86 |
| Deck C | 0.008 ± 0.0.27 | 0.062 ± 0.0.31 | 0.68 |
| Deck D | 0.056 ± 0.28 | 0.11 ± 0.32 | 0.52 |

Data are presented as means ± standard deviations for the male and female older adults. An independent samples t-test was used to examine the gender differences in good choice, bad choice, good vs. bad, and total income. A mixed ANOVA was used to examine the gender effect on the IGT performance. For the proportion of choices, there was no significant main effect of gender (F(1,49) = 2.56, p = 0.12), or interaction effect between gender and deck (F(1,49) = 0.60, p = 0.44). For the CDS, there was no significant main effect of gender (F(1,49) = 0.06, p = 0.81), or interaction effect between gender and deck (F(1,49) = 3.12, p = 0.08). In the table, Post hoc tests were used to compare males and females for each deck (FDR corrected p < 0.05). Abbreviations: IGT, Iowa gambling task; CDS, change of deck selection.

**Supplementary Table 3. Correlations between age-related activation and CDS.**

| **Region** | **CDS** | **P value** |
| --- | --- | --- |
| *Deck A* |  |  |
| right SMA | 0.22 | 0.17 |
| Right IPL | 0.29 | 0.068 |
| Left MFC | **0.39** | **0.018** |
| right MFC | 0.30 | 0.076 |
| Right SMFC | **0.41** | **0.024** |
| right Mid_OFC | 0.21 | 0.16 |
| *Deck B* |  |  |
| Med_OFC | 0.28 | 0.17 |
| Right Mid_OFC | 0.19 | 0.20 |
| LG | 0.22 | 0.21 |

After extracting the values of activation within the significant brain regions, partial correlations were conducted to examine the correlations between CDS and brain activations in decks A and B, controlled for MoCA, head motion, and brain atrophy (FDR corrected p < 0.05). Abbreviations: CDS, change of deck selection; IPL, inferior parietal lobe; SMFC, superior medial frontal cortex; LG, lingual gyrus; MFC, middle frontal cortex; Mid_OFC, middle orbitofrontal cortex; Med_OFC, medial orbitofrontal cortex; SMA, supplementary motor area.

**Supplementary Figure 1.** Correlation patterns between brain activation and age in the IGT. For each deck, Partial correlations were conducted to examine the relationships between brain activation and age, controlled for MoCA, brain atrophy, and head motion (FDR corrected p < 0.05). The heat map of partial correlation coefficients exhibits that age-effect was observed mainly in disadvantageous decks relative to advantageous decks. Values in heat map indicated the correlation coefficients with significant relationships highlighted in white color.


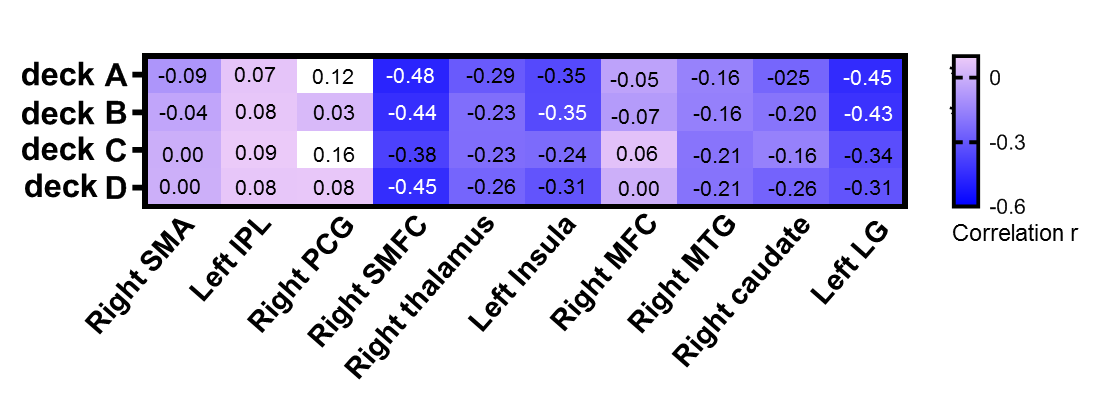


**Supplementary Figure 2.** Correlation analyses without controlling for head motion. Head motion has been known to have confounding effects on task-related brain activity, and usually is controlled as a covariate of non-interest in statistical analysis. Pearson correlation showed that age was positively correlated with head motion (r = 0.42, p = 0.002). To further examine how head motion affected age-effect on brain activations corresponding to the four decks, partial correlations were conducted without controlling for head motion. **A)** The heat map of partial correlation coefficients was consistent with the results in Figure 3B, showing larger age-effect in disadvantageous decks relative to advantageous decks (FDR correction). *, p < 0.05; **, p < 0.01; ***, p < 0.005. **B)** The voxel-based whole brain analyses showed significant negative correlations between age and brain activation in disadvantageous decks, but not in advantageous decks, showing consistent patterns with the results in **Figure 4A**.


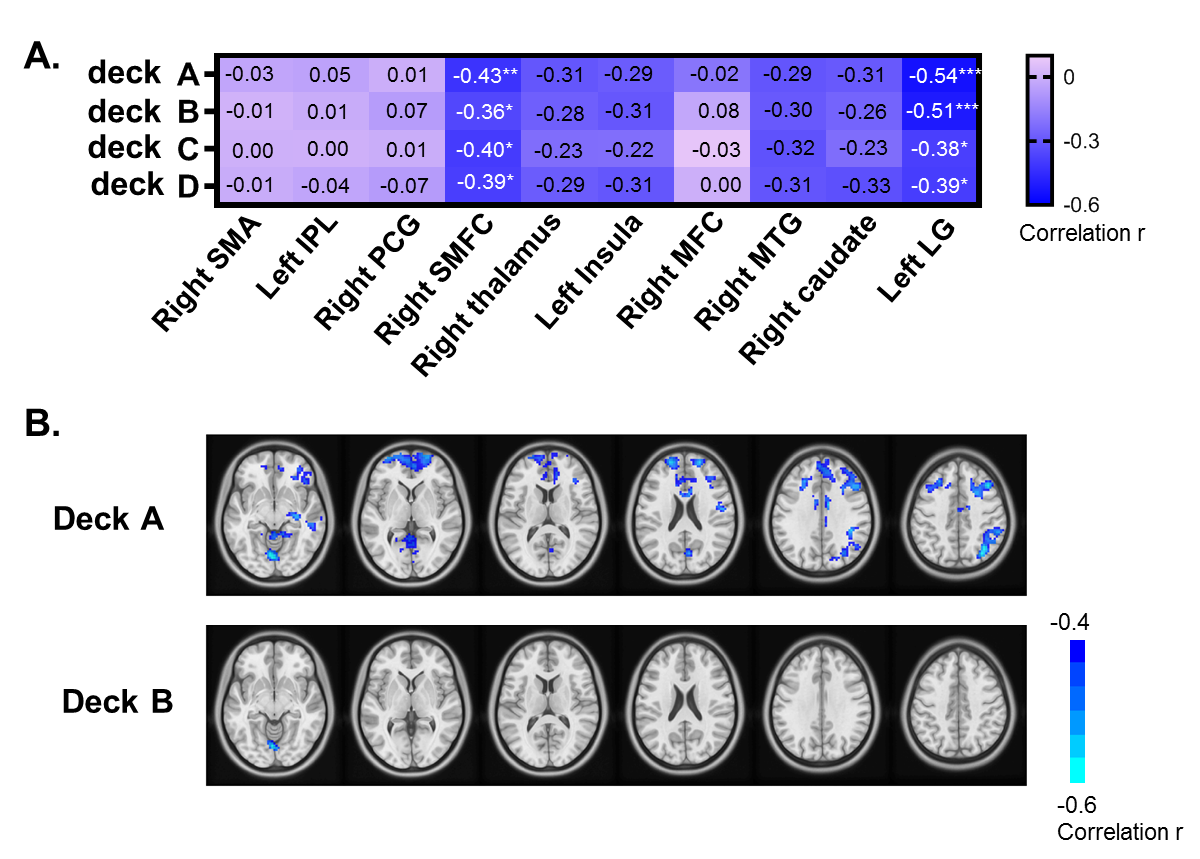


**Supplementary Figure 3.** The differences of brain activation between each pair of decks. A paired t-test was used to compare brain activations between different decks. The threshold of FDR corrected p < 0.008 (0.05/6) and cluster > 50 voxels was used due to six comparisons. The resulting brain maps showed significant brain regions consistent with the findings in the one-way repeated measures ANOVA.


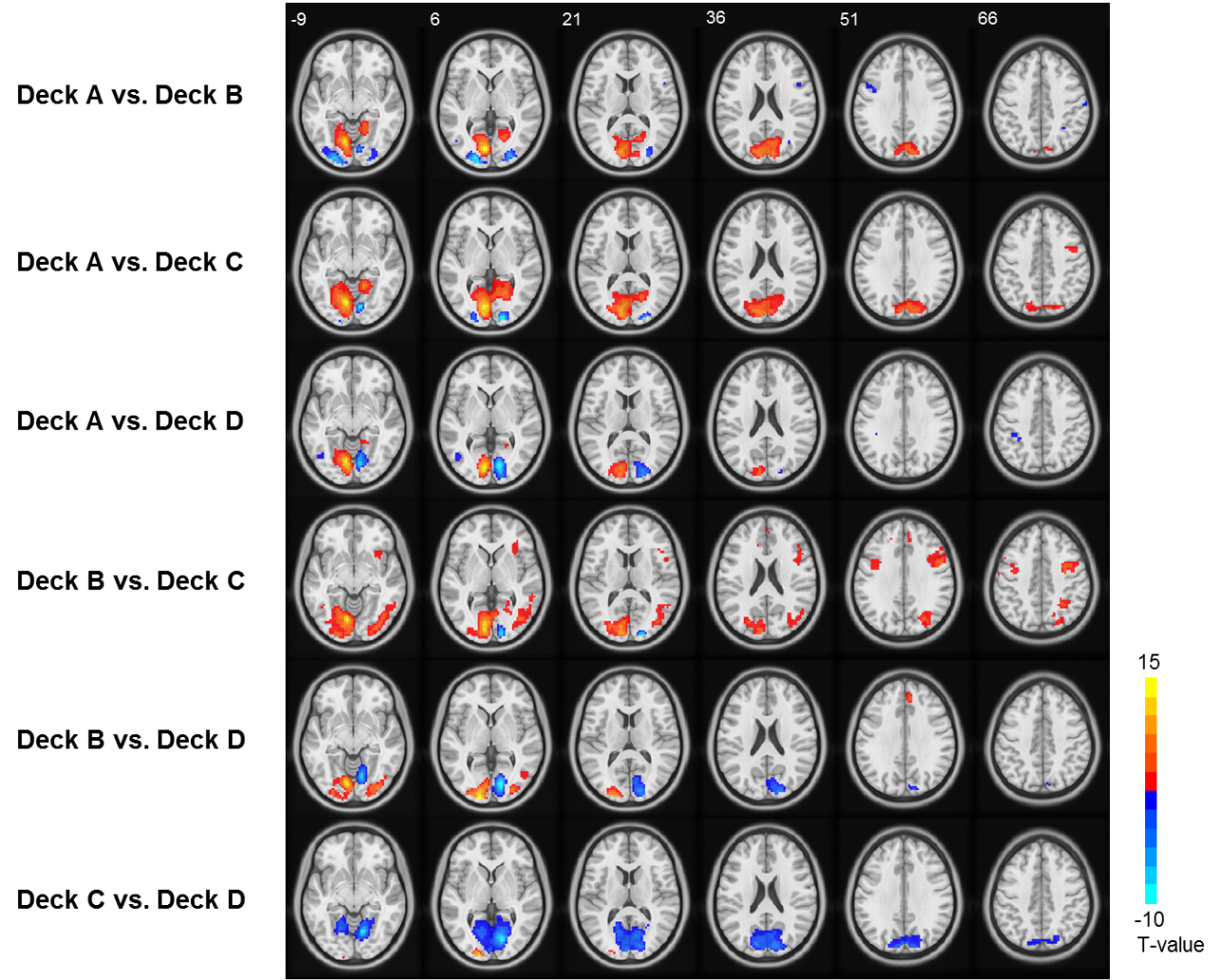

Supplement: Supplementary file 1 [file Data_Sheet_1.docx]
